# Oxytocin ameliorates impaired social behavior in a *Chd8* haploinsufficiency mouse model of autism

**DOI:** 10.1186/s12868-021-00631-6

**Published:** 2021-05-01

**Authors:** Stanislav M. Cherepanov, Maria Gerasimenko, Teruko Yuhi, Kazumi Furuhara, Chiharu Tsuji, Shigeru Yokoyama, Keiichi I. Nakayama, Masaaki Nishiyama, Haruhiro Higashida

**Affiliations:** 1 Department of Basic Research on Social Recognition and Memory, Research Center for Child Mental Development, Kanazawa University, Kanazawa, 920-8640 Japan; 2 Department of Molecular and Cellular Biology, Medical Institute of Bioregulation, Kyushu University, Fukuoka, 812-8582 Japan; 3 Department of Histology and Cell Biology, Graduate School of Medical Sciences, Kanazawa University, Kanazawa, 920-8640 Japan

**Keywords:** Autism, Oxytocin, CHD8, Social novelty, Anxiety

## Abstract

**Background:**

Autism spectrum disorder (ASD) is characterized by the core symptoms of impaired social interactions. Increasing evidence suggests that ASD has a strong genetic link with mutations in chromodomain helicase DNA binding protein 8 (*CHD8*), a gene encoding a chromatin remodeler. It has previously been shown that *Chd8* haplodeficient male mice manifest ASD-like behavioral characteristics such as anxiety and altered social behavior. Along with that, oxytocin (OT) is one of the main neuropeptides involved in social behavior. Administration of OT has shown improvement of social behavior in genetic animal models of ASD. The present study was undertaken to further explore behavioral abnormalities of *Chd8* haplodeficient mice of both sexes, their link with OT, and possible effects of OT administration. First, we performed a battery of behavioral tests on wild-type and *Chd8*^+/∆SL^ female and male mice. Next, we measured plasma OT levels and finally studied the effects of intraperitoneal OT injection on observed behavioral deficits.

**Results:**

We showed general anxiety phenotype in *Chd8*^+/∆SL^ mice regardless of sex, the depressive phenotype in *Chd8*^+/∆SL^ female mice only and bidirectional social deficit in female and male mice. We observed decreased level of OT in *Chd*^+/∆SL^ mice, possibly driven by males. Mice injected by OT demonstrated recovery of social behavior, while reduced anxiety was observed only in male mice.

**Conclusions:**

Here, we demonstrated that abnormal social behaviors were observed in both male and female *Chd8*^+/∆SL^ mice. The ability of peripheral OT administration to affect such behaviors along with altered plasma OT levels indicated a possible link between Chd8 + /∆SL and OT in the pathogenesis of ASD as well as the possible usefulness of OT as a therapeutic tool for ASD patients with* CHD8* mutations.

**Supplementary Information:**

The online version contains supplementary material available at 10.1186/s12868-021-00631-6.

## Background

Chromodomain helicase DNA binding protein 8 (CHD8) is a member of the chromodomain helicase DNA-binding (CHD) family of proteins, and functions as an ATP-dependent chromatin-remodeling factor in the regulation of expression of many genes, including those for β-catenin and p53 [1–3]. *CHD8* generates two alternatively spliced transcripts that encode a full-length 280-kDa protein (CHD8_L_) or 110-kDa protein (CHD8_S_) that contains only the NH_2_-terminal chromodomain [4, 5]. Mutations identified in individuals with autism spectrum disorder (ASD) are distributed throughout the *CHD8* locus, with some predicted mutations resulting in the loss of both CHD8 isoforms, while others affect only CHD8_L_ [4, 6–8]. To recapitulate this situation, we previously generated two independent lines of mutant mice lacking either both CHD8 isoforms (ΔSL) or only CHD8_L_ (ΔL) [4]. Mice homozygous for either mutation die in utero, whereas heterozygous mutant mice (*Chd8*^+/∆SL^) are viable [4, 9–11], and have been shown to be a representative mouse model of ASD.

It has been reported that mice carrying heterozygous mutations or gene knockdown of *Chd8* display various autism-like phenotypes [4, 12–15], such as macrocephaly, social deficits, repetitive behavior, and cognitive impairments [10]. Some researchers have produced mice carrying a heterozygous mutation in *Chd8* (*Chd8*^+/N2373K^) that reflects an identical mutation found in patients carrying a human *CHD8* mutation (Asn2373LysfsX2). Male *Chd8*^+/N2373K^ mice display an abnormality in maternal-dependent ultrasonic vocalization (USV) changes that are not observed in female mice. However, deletion of *Chd8* is associated with abnormal activation of the RE-1 silencing transcription factor, which suppresses the transcription of many neuronal genes in both sexes [4].

Oxytocin (OT) is a nonapeptide hormone, which was originally identified as part of the female reproductive process [16, 17]. However, many studies now indicate that OT is a fundamental molecule for social behavior [18–23]. In humans, it has been reported that OT administration promotes social cognition and prosociality in interpersonal relationships among typically developing individuals [24–28]. Consequently, the results of these studies led to the notion that OT may be beneficial for patients with the core symptoms of ASD [29–34]. Although no marked improvements have been reported, increased social interaction was recognized by caregivers or family members, and subjective scores for judging ASD phenotypes showed encouraging results in only the social domain [32, 35].

Using *Chd8* haploinsufficiency (*Chd8*^+/∆SL^) mice, we previously reported male phenotypes [4]. In the current experiment, we address two questions: whether impaired social behavior is observed in female *Chd8*^+/∆SL^ mice (because the information is lacking in knockout female mice); and whether abnormal social behavior in male and female *Chd8*^+/∆SL^ mice can be recovered by OT administration. The reason being that if such phenotypes are sensitive to OT treatment, as in CD38 or CD157 knockout mice [18, 36–41], a similar approach may be therapeutically beneficial for patients with ASD associated with *CHD8* mutations.

## Results

*Chd8*^+/∆SL^ male mice manifested increasing anxiety-like behavior in a range of behavioral tests, which partially replicates findings in patients with ASD [4]. Because this is such an important point, we further investigated and compared social behavior in male and female mice.

### Behavior during open field exposure and two-phase social avoidance test

**Fig. 1:**
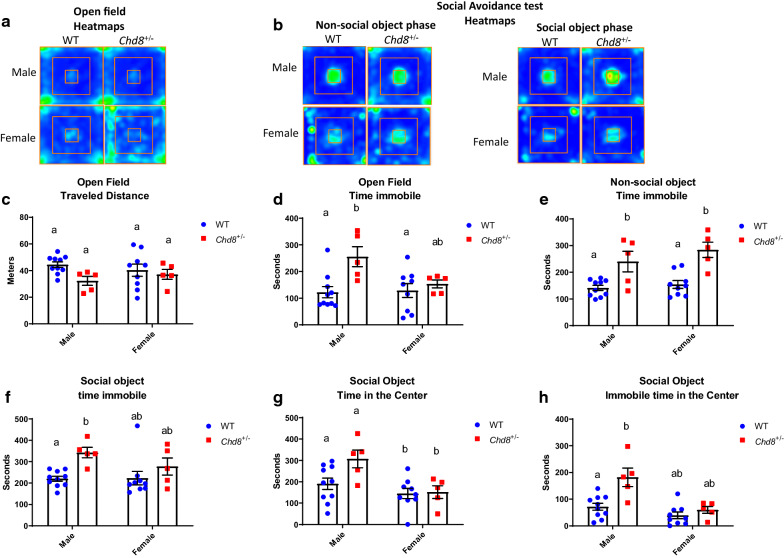
The behavior of wild-type (WT, blue) and *Chd8*^+/∆SL^ (*Chd8*^±^, red) mice in the open field test and social avoidance test. **a** Cumulative heatmaps for Open field (**b**) and non-social object and social object phases of Social Avoidance task. **c** Distance traveled during the duration of Open Field test. **d** Immobility time during the duration of Open Field test. **e** Immobility time in the “non-social object” stage. **f** Immobility time in the “social object” stage. **g** Time spent in the center in the social-object stage. **h** Immobility time in the center in the social-object stage. Columns with different superscript letters (**a**, **b**) are significantly different at *p* < 0.05

We examined behavior in the open field and social avoidance test. In the open field and both phases of the avoidance test (with a non-social object and social object), no differences in locomotion were observed (Fig. 1a–c; Additional file 1: Fig. S1c, f). These data indicate no obvious defect in physical ability, such as locomotion, in both sexes of *Chd*^+/∆SL^ mice.

While in such parameters like time in center and immobile time in center in the Open Field we did not observe differences (Additional file 1: Fig. S1a, b). Two-way ANOVA showed a significant effect of genotype (F (1, 25) = 8.480 P = 0.0075) and tendency of effect for sex (F (1, 25) = 3.127, P = 0.0892) and interaction (F (1, 25) = 4.036, P = 0.0555) for immobility time (Fig. 1d). WT male mice were immobile significantly shorter time (122.3 ± 20.8 s, n = 10) than *Chd*^+/∆SL^ male mice (255.3 ± 37.7 s, n = 5; p < 0.05 by multiple comparisons followed Bonferroni correction).

We observed mouse behavior to a non-social target in the center zone in the first stage of social avoidance test. Genotype difference for immobility time was found (Fig. 1b, e). Two-way ANOVA showed a significant effect of genotype (F (1, 25) = 30.62, P < 0.0001 and no effect for sex (F (1, 25) = 1.897, P = 0.1806) or interaction (F (1, 25) = 0.5666, P = 0.4586). Consistently, immobility time was significantly shorter in WT male mice (141.3 ± 9.7 s, *n* = 10) than in *Chd8*^+/∆SL^ male mice (240.2 ± 38.5 s, *n* = 5); p < 0.05, and immobility time of WT female mice (154.2 ± 14.5 s, n = 9) was shorter than in *Chd8*^+/∆SL^ female mice (284.2 ± 28.7 s, *n* = 5); *p* < 0.05; Fig. 1e).

At the final step, with a social target mouse placed in the center zone, like in the previous phase, the effect of genotype only on immobility time was observed: two-way ANOVA for genotype, F (1, 25) = 10.06, P = 0.0040. Immobility time was significantly shorter in WT male mice (220.4 ± 11.6 s, *n* = 10) than in *Chd8*^+/∆SL^ male mice (342.8 ± 24.4 s, n = 5); *p* < 0.05, Fig. 1f. The same inclination was demonstrated by females: immobility time of WT female mice (223.2 ± 31.5 s, *n* = 9) was significantly shorter than *Chd8*^+/∆SL^ female mice (284.2 ± 28.7 s, *n* = 5); *p* < 0.05, Fig. 1f.

Time spent in the center zone was different only between sexes regardless of genotype. Two-way ANOVA for sex (F (1, 25) = 10.55, P = 0.0033). The difference in time spent in the center zone was significant between male and female WT and *Chd8*^+/∆SL^ mice; *p* < 0.05, Fig. 1g. For immobility time in the center zone effect of genotype, sex and interaction were significant. Two-way ANOVA for genotype (F (1, 25) = 10.06, P = 0.0040), sex (F (1, 25) = 18.13, P = 0.0003) and interaction (F (1, 25) = 6.113, P = 0.0206). Immobility time in the center area in the presence of a social target was significantly shorter in WT male mice (71.5 ± 13.0 s, *n* = 10) than in *Chd8*^+/∆SL^ male mice (182.1 ± 34.6 s, *n* = 5) (Fig. 1h).

Altogether, these results (Fig. 1) indicate that anxiety-like behavior is different between genotypes, while social avoidance-like behavior is different between sexes rather than by genotype.

### Social behavior in the three-chamber test

**Fig. 2:**
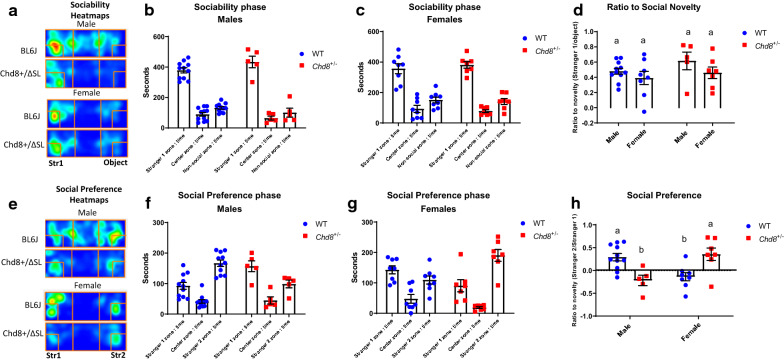
Social novelty preference behavior of wild-type (WT, blue) and *Chd8*^+/∆SL^ (*Chd8*^+/−^, red) mice in the three-chamber test. In the sociability phase, bars indicate time spent in the left chamber with a social target (stranger 1), while in the empty center chamber and right chamber with a non-social target for male (**a**) and female (**b**) mice. **c** The ratio for novelty in sociability phase (time with stranger 1/non-social object). In the social preference phase, bars indicate time spent in the left chamber with a familiar mouse (stranger 1), while in the empty center chamber and left chamber with a new mouse (Stranger 2) for male (**d**) and female (**e**) mice. **f** The ratio for novelty (time with Stranger 2 / time with Stranger 1). Columns with different superscript letters (**a**, **b**) are significantly different at *p* < 0.05

To examine more precisely the phenotypes of abnormal sociability and social novelty preference in *Chd8*^+/∆SL^ mice, we used a three-chamber box test. For the first experiment, a target male mouse was placed in the left chamber (stranger 1), in which the sociability phenotype (preference of a social than non-social target) can be measured. Time spent with Stranger 1 was longer compared with the non-social target equally in males of both genotypes (378.5 ± 16.9 s vs 131.5 ± 8.8 s, *n* = 11 for WT and 433.9 ± 37.9 s vs 100.8 ± 28.8 s, *n* = 5 for *Chd8*^+/∆SL^; Fig. 2a, b). A similar preference to the social target was observed in female mice of both genotypes (356.6 ± 32.7 s vs 151.0 ± 18.5 s, *n* = 8 for WT and 381.4 ± 21.5 s vs 141.0 ± 19.5 s, *n* = 7 for *Chd8*^+/∆SL^ Fig. 2c). The ratio for social novelty was calculated (Fig. 2d). Two-way ANOVA did not show significance for sex (F (1, 27) = 2.665, P = 0.1142), genotype (F (1, 27) = 1.869, P = 0.1828) or interaction (F (1, 27) = 0.1994, P = 0.6588). Thus, *Chd8*^+/∆SL^ mice of both sexes possess normal sociability, by preference social stimulus over non-social.

When a second target mouse was placed in the right chamber, time spent with the new social target (Stranger 2) was higher compared with the chamber containing a familiar mouse (stranger 1) in WT male mice (92.5 ± 11.5 s vs 166.7 ± 10.4 s, *n* = 11; Fig. 2e, f). Surprisingly, such social novelty preference (to stranger 2) was lost in *Chd8*^+/∆SL^ male mice (156.9 ± 17.9 s vs 98.7 ± 12.4 s, *n* = 5; Fig. 2f). Although social preference was less clear cut in WT female mice (142.9 ± 12.9 s vs 109.5 ± 13.8 s, *n* = 8; *p* = 0.31; Fig. 2g), *Chd8*^+/∆SL^ female mice showed a preference to stranger 2 (90.7 ± 19.7 s vs 190.3 ± 19.6 s, *n* = 7; Fig. 2g).

Next, we calculated the ratio index for social preference (Fig. 2h). A positive ratio (preference to novelty over familiar mice) was apparent in WT male (0.29 ± 0.08) and *Chd8*^+/∆SL^ female (0.36 ± 0.14) mice, while negative values were obtained for *Chd8*^+/∆SL^ male (− 0.22 ± 0.11) and WT female (− 0.14 ± 0.1) mice. Two-way ANOVA indicated significant difference for sex × genotype interaction (F (1, 27) = 21.68, P < 0.0001). Therefore, multiple comparisons followed by Bonferroni correction were performed and indicated a significant difference of ratio for WT male from thus of ratio for *Chd8*^+/∆SL^ male and WT female as well as the difference of ratio for *Chd8*^+/∆SL^ female from WT female and *Chd8*^+/∆SL^ male.

These results indicate a lower social preference in *Chd8*^+/∆SL^ male mice and higher social preference in *Chd8*^+/∆SL^ female mice, which are two distinct abnormal phenotypes compared with the phenotype in WT mice of both sexes.

### Behavior during the light–dark transition test

Anxiety-like behavior was further examined using two variations of a light–dark transition test. In the first variation, mice were initially placed in the light zone at the start of experiments, while in the second “reverse” variant test, they were first placed in the dark zone.

**Fig. 3:**
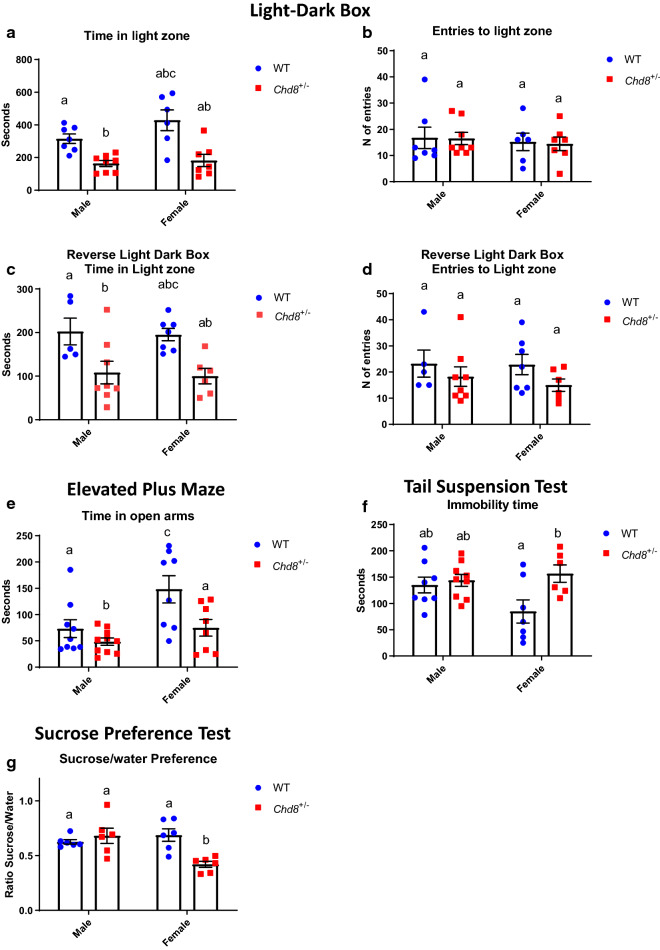
Anxiety and depressive-like behavior of wild-type (WT, blue) and *Chd8*^+/∆SL^ (*Chd8*^±^, red) mice. The light–dark box (**a**–**d**). A test mouse started from the light zone. **a** Time spent in the light zone. **b** The number of entries to the dark zone. Reverse tests initiated from the dark zone. *n* = 5–7. **c** Time spent in the light zone when started from the dark zone. **d** The number of entries. **e** Elevated plus maze test. Bars represent time spent in the open arm for a 5-min period. **f** Tail suspension test. Bars represent immobility time. **g** Sucrose preference test. Bars represent the consumption ratio of 1% sucrose over water. Columns with different superscript letters (**a**–**c**) are significantly different at *p* < 0.05

When mice were first placed in the light arena, the time spent in the light zone was significantly shorter in *Chd8*^+/∆SL^ male mice (163.9 ± 18.3 s, *n* = 8) than WT male mice (315.6 ± 28.6 s, *n* = 7; *p* < 0.05; Fig. 3a). Time spent in the light zone was also significantly shorter in *Chd8*^+/∆SL^ female mice (182.1 ± 37.3 s, *n* = 7) than WT female mice (429.0 ± 64.3 s, *n* = 6; *p* < 0.05; Fig. 3a). Two-way ANOVA indicated significance of genotype only (F (1, 24) = 28.03, P < 0.0001), but not sex (F (1, 24) = 3.057, P = 0.0932) or interaction (F (1, 24) = 1.595, P = 0.2188).

The number of entries was not different among any groups of *Chd8*^+/∆SL^ or WT male and female mice (Fig. 3b). This suggests that *Chd8*^+/∆SL^ mice prefer to stay in the dark after starting in the light zone, i.e., anxiety is higher in *Chd8*^+/∆SL^ regardless of sex.

Reversely, when the mice were first placed in the dark arena prior to starting the experiments, the time spent in the light zone in *Chd8*^+/∆SL^ male mice (108.1 ± 26.0 s, *n* = 8) was significantly shorter compared with WT male mice (202.3 ± 30.8 s, *n* = 5, *p* < 0.05; Fig. 3c). The same was found for female mice (99.9 ± 17.8 s, *n* = 6 for *Chd8*^+/∆SL^ vs 195.2 ± 14.1 s, *n* = 7 for WT; *p* < 0.05; Fig. 3c). Two-way ANOVA indicated significance of genotype, (F (1, 24) = 28.03, P = 0.0001), but not sex (F (1, 24) = 3.057, P = 0.0932) or interaction (F (1, 24) = 1.595, P = 0.2188). The number of transitions in both male and female *Chd*8^+/∆SL^ mice was unchanged as well as between male and female WT mice (Fig. 3d). These results also show that *Chd8*^+/∆SL^ mice stay longer in the dark box, possibly due to anxiety.

### Elevated plus maze test

Next, we used an elevated plus maze test, the most standardized experiment for measuring anxiety in a mouse model. Using time in open arms as primary outcome measure, Two-way ANOVA showed a significant effect of genotype (F (1, 31) = 9.024, P = 0.0052) and sex (F (1, 31) = 8.422, p = 0.0068) but not interaction (F (1, 31) = 2.083, P = 0.1590, n = 8–10.). As shown in Fig. 3e, WT male mice (73.2 ± 16.8 s, *n* = 9) spent longer time in the open arm than male *Chd8*^+/∆SL^ mice (48.7 ± 7.1 s, *n* = 10). A similar difference was observed in females: WT female mice (148.2 ± 25.9 s, *n* = 8) spent longer time in the open arm compared to *Chd8*^+/∆SL^ female mice (75.0 ± 15.8 s, *n* = 8). This data indicates that male mice spent less time in open arms than female and WT mice spent more time in open arms than *Chd8*^+/∆SL^ mice. This data indicate *Chd8*^+/∆SL^ mice possibly demonstrate more anxious phenotype.

### Tail suspension test

Next, we examined depression-like or hyperactive behavior using the tail suspension test. For immobility time, Two-way ANOVA showed a significant effect of genotype (F (1, 26) = 6.154, P = 0.0199) no effect for sex (F (1, 26) = 1.315, P = 0.2619) and tendency for interaction (F (1, 26) = 3.772, P = 0.0630), n = 6–9. According to post-hoc tests, immobility time was significantly longer in female *Chd8*^+/∆SL^ mice (156.8 ± 16.5 s, *n* = 7) than in WT female mice (84.9 ± 22.2 s, *n* = 7; *p* < 0.05; Fig. 3f) suggesting genotype difference possibly due to *Chd8*^+/∆SL^ female mice, that show depressive-like behavior.

### Sucrose preference test

Subsequently, we performed another test for depression and anhedonia in mice, specifically, the two-bottle choice test (containing water or 1% sucrose solution). Two-way ANOVA showed a significant effect of genotype (*F* (1, 20) = 4.932, P = 0.0381) and interaction (*F* (1, 20) = 11.28, P = 0.0031). That data indicated genotype difference depended on the sex of the animal. Bonferroni multiple comparisons indicate that *Chd8*^+/∆SL^ mice (0.42 ± 0.03, *n* = 6) displayed lower sucrose preference than WT female mice (0.69 ± 0.06, *n* = 6); *p* < 0.05; Fig. 3g), suggesting that *Chd8*^+/∆SL^ female mice lose hedonic interest compare to wild type female and genotype difference does not exist in males.

### Role of OT in *Chd8* haploinsufficiency mice

#### Maternal behavior

Using three pups each of WT and *Chd8*^+/∆SL^ genotypes, we examined parental (retrieval) behavior of the first mother (primiparous dams) at postpartum day 3 for five postnatal days in three trials. *Chd8*^+/∆SL^ dams displayed retrieval behavior as rapidly as WT dams, with no statistical difference found (*n* = 5; Additional file 2: Fig. S2). This suggests that *Chd8*^+/∆SL^ dams possess the same motherhood capabilities as WT dams.

#### Plasma OT levels

**Fig. 4:**
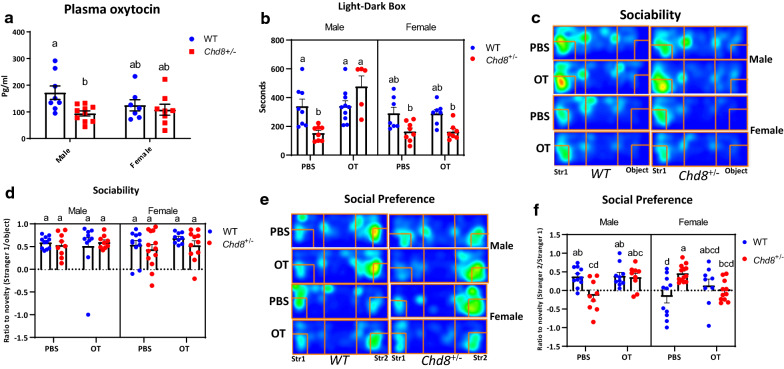
Role of oxytocin in *Chd8*^+/∆SL^ mice. **a** The concentration of oxytocin (OT) in deproteinized plasma of wild-type (WT) and *Chd8* (*Chd8*^±^) mice. **b** Effect of saline (PBS) or OT on the behavior of WT and *Chd8*^+/∆SL^ mice in the light–dark transition test starting from the light zone. **b** Time spent in the light zone. **c–f** Effect of saline (PBS) or OT on the behavior of WT and *Chd8*^+/∆SL^ mice in the three-chamber box test. Cumulative heatmaps for sociability phase (**c**), the ratio for social novelty (**d**), cumulative heatmaps for social preference phase (**e**), and ratio for social preference (**f**). Columns with different superscript letters (**a**–**d**) are significantly different at *p* < 0.05

To understand the reason for the impaired social behavior of *Chd8*^+/∆SL^ mice, we measured plasma OT levels. Two-way ANOVA followed by Bonferroni Post-hoc tests showed a significant effect of genotype (F (1, 29) = 6.092, P = 0.0197) but not sex F (1, 29) = 0.7529, P = 0.3927) and low tendency for interaction (F (1, 29) = 2.567, P = 0.1200, n = 7–10). Plasma concentration of OT was significantly lower in *Chd8*^+/∆SL^ male mice (94.1 ± 9.6 pg/ml, *n* = 10) compared with WT male mice (172.2 ± 25.4 pg/ml, *n* = 8; *p* < 0.05; Fig. 4a). While no difference was observed between females. However, limited sample size not allowed us, to conclude that observed genotype differences are sex depended.

#### Recovery of impaired behavior by OT

Accordingly, the next question we asked is whether this impaired social behavior can be ameliorated by the rescue of plasma OT levels.

Based on our plasma OT results, we determined whether OT has any beneficial effects on impaired social behavior in *Chd8*^+/∆SL^ mice. We used the light–dark transition test, where, twenty minutes after i.p. injection of OT (100 ng/100 g of body weight) or saline, male or female mice were initially placed in the light zone. After completing these tests, we performed three-way ANOVA, using time in light area as primary outcome, for investigation of sex, genotype and treatment effects. Significance was observed for all three factors: treatment (F (1, 52) = 8.876, P = 0.0044) genotype (F (1, 52) = 7.769 P = 0.0074) and sex (F (1, 52) = 13.91, P = 0.0005) as well as interactions of factors: genotype × sex (F (1, 52) = 8.819, P = 0.0045), treatment × sex (F (1, 52) = 9.193, P = 0.0038) and interactions of all three factors (F (1, 52) = 8.878, P = 0.0044). After multiply comparisons, essentially no effect was observed on time spent in the light zone in WT male mice (339.4 ± 50.1 s, *n* = 8) for saline treated mice vs 341.0 ± 36.8 s, *n* = 10 for OT treatment; *p* = 0.99; Fig. 4b). Contrarily, in *Chd8*^+/∆SL^ male mice, time spent in the light zone became significantly longer in OT-treated mice (being comparable to WT levels) (476.8 ± 74.3 s, *n* = 5) compared with knockout phenotype levels (153.1 ± 17.9 s, *n* = 8, phosphate-buffered saline [PBS]-treated; *p* < 0.05).

In contrast, as expected, OT had no effect on time spent in the light zone in female WT mice (290.0 ± 42.9 s, *n* = 7 for OT vs 288.8 ± 27.9 s, for saline, *n* = 7; *p* = 0.99) or *Chd8*^+/∆SL^ mice (164.7 ± 23.7 s, *n* = 8 for OT vs 163.0 ± 21.8 s, *n* = 7 for saline; *p* = 0.99; Fig. 4b). These results indicate that while OT has effect on *Chd8*^+/∆SL^ genotype and has an essential effect in *Chd8*^+/∆SL^ male mice only in the light–dark transition test.

Next, we evaluated effects of OT on the sociability and social preference. We used the three-chamber test, where, twenty minutes after i.p. injection of OT (100 ng/100 g of body weight) or saline, male or female mice were placed to the chamber. The time spent in each chamber in the sociability and social preference steps with saline or OT treatment in WT and *Chd8*^+/∆SL^ mice is shown in (Additional file 3: Fig. S3; Fig. 4c, e). Three-way ANOVA for the ratio to social novelty in the sociability phase did not show any significance for sex (F (1, 75) = 0.02675, P = 0.8705), genotype (F (1, 75) = 0.6555, P = 0.4207), treatment (F (1, 75) = 0.6243, P = 0.4320) or interaction of these factors (Fig. 4d). This data indicated the absence of OT effect on sociability of both WT and *Chd8*^+/∆SL^ mice of both sexes.

Three-way ANOVA for social preference ratio in last phase indicated significance for interactions of factors. Treatment × sex (F (1, 75) = 5.024, P = 0.0280), genotype × sex (F (1, 75) = 8.577, P = 0.0045), and interaction of all three factors (F (1, 75) = 13.51, P = 0.0004). Interestingly, in *Chd8*^+/∆SL^ male mice, interest to a new social target was recovered from saline-treated levels (− 0.15 ± 0.14, *n* = 9) by OT treatment (0.36 ± 0.10, *n* = 9; *p* < 0.05; Fig. 4f), and was identical to levels in WT male mice treated with saline (0.31 ± 0.08, *n* = 11) or OT (0.38 ± 0.10, *n* = 10, *p* = 0.99). Regarding female mice (Fig. 4f), a higher Str 2/Str 1 ratio in *Chd8*^+/∆SL^ mice (0.46 ± 0.06, *n* = 13 for PBS) was corrected to levels of no preference (− 0.01 ± 0.09, *n* = 11 for PBS; *p* < 0.05). In WT female mice, OT treatment reversed social preference from no preference (− 0.17 ± 0.17, *n* = 11 for saline; *p* = 0.99) to a slight preference (0.13 ± 0.17, *n* = 9).

## Discussion

Here, we show that abnormal social behaviors are displayed in both male and female *Chd8*^+/∆SL^ mice (Table 1). *Chd8*^+/∆SL^ mice exhibited anxiety-like behavior in three different tests. Meanwhile, female *Chd8*^+/∆SL^ mice exhibited depression-like behavior. Therefore, our first finding is that defects in social behavior are not limited to male knockout mice but are also present in female *Chd8*^+/∆SL^ mice. Our results in male mice are consistent with a previous report by Katayama et al. [4]. Alternatively, the impaired behavior observed here in females, indicates for the first time at least that defects in social behavior may not be preponderant in male mice [10].

**Table 1:** Summary of behavioral tests, oxytocin concentrations and effects of oxytocin in *Chd8*^+/∆SL^ mice

Parameter	Test	Phenotype	Oxytocin effect
Locomotion	Open field	Normal	
Social Avoidance	Social avoidance test	Normal	
Anxiety	Social avoidance test Elevated plus maze Light dark box Reverse light dark box	Anxious	Corrected in males in light dark box
Sociability	3-chamber box	Normal	
Social preference	3-chamber box	Absence in males Increased to novelty in females	Corrected
Depression	Sucrose preference Tail suspension test	Depressive phenotype in females	
Oxytocin concentration		Decreased in males	

Social behavioral impairments in female *Chd8*^+/∆SL^ mice are indicative of the behavioral defects of female patients with ASD due to *CHD8* mutation because ASD associated with *CHD8* mutation is not limited to male patients [44]. All of the social behavioral impairments observed in female *Chd8*^+/∆SL^ mice using multiple paradigms suggests the presence of psychiatric traits in patients with ASD because the multiple tasks applied to *Chd8*^+/∆SL^ mice are well-established methods for animal models of human psychiatric disorders [45].

In the open field test, *Chd8*^+/∆SL^ male mice spent the same time in the center zone as WT mice in a novel environment without any targets, which replicates previous reports [14, 15], but are inconsistent with another report by Gompers et al. [13] in *Chd8* mutant mice. Katayama et al. [4], Platt et al. [14], and Suetterlin et al. [15] showed that *Chd8* haploinsufficient male mice were hypermobile, while we observed no difference in traveled distance and increased immobility time in *Chd8*^+/∆SL^ male mice (Fig. 1d). However, this may not be contradictory, because the exact protocols of the test were different. While Katayama et.al. used 120 min for open field, we used 10 min. In the social avoidance task, in both phases, in presence of the non-social or social object, regardless of sex, immobility time was increased in *Chd8*^+/∆SL^ mice.

Judging from the pattern of sociability [45] in the three-chamber test, sociability was not disrupted in both sexes of *Chd8*^+/∆SL^ mice. However, social novelty preference was lost in *Chd8*^+/∆SL^ male mice. Interestingly, this deficit in male mice was in part ameliorated by intraperitoneal injection of OT, after that male mice showed interest in a new social target. Unexpectedly, *Chd8*^+/∆SL^ female mice stayed with the new target mouse, resembling the behavior of WT males. In contrast to our data, previous reports [46, 47] indicated a social preference for novel social stimulus, for C57BL/6 female mouse. However, most of these works used a same-sex approach for males and opposite-sex stimuli for females, while we used same-sex stimuli in both cases, which can lead to different observed phenotype in wild-type mice.

After OT treatment, abnormal phenotype disappeared in *Chd8*^+/∆SL^ females by an unknown mechanism, while phenotype of WT females remains (Table 1). Such changes in *Chd8*^+/∆SL^ females seem to be owing to an indirect effect, although it is known that OT can influence social preference in female mice in specific conditions [48, 49]. Additionally, while we measured peripheral OT levels, it might be more important to determine the OT level in the cerebrospinal fluid and brain tissue. A limitation of this study is that we did not calculate time in chambers, during the habituation phase. However, while saline and OT treated Chd*8*^+/∆SL^ mice demonstrated different preferences in the last phase of ties with unknown place preference in the habituation phase, previous reports indicated, OT itself can not alter place preference [48].

During the social-avoidance task, in presence of the social object, females of both genotypes spent less time in the center compared with males. Hence, we can conclude ,that there observed no social anxiety in *Chd8*^+/∆SL^ mice, while their immobility time was increased in the context of the novel environment. Such novelty of presented object (but not altered traveled distance) can suggest about generally anxious genotype. An important limitation of these tests: while using ANOVA we observe genotype difference in immobility time all stages of tests, multiply comparisons followed by corrections indicated the difference in all stages only for males but only for the stage with the presented non-social object in females. Therefore, conclusions about anxious phenotype require to consider other tests.

The light–dark transition test is a straightforward test to determine how keen a mouse is to move from an original compartment into a new light or dark environment [50]. In the current experiments, we replicated previously observed deficits in anxiety [4, 14]. Our novel finding, however, is that *Chd8*^+/∆SL^ female mice spent longer in the dark, a sign of anxiety. These findings were replicated, regardless of the starting point (light or dark zone). And also, were confirmed by the results of the elevated plus maze. Overall data suggesting anxious phenotype in *Chd8*^+/∆SL^ mice regardless of sex. More importantly was finding that when *Chd8*^+/∆SL^ male mice were treated by i.p. injection of OT, they stayed longer in the light compartment (like WT male mice), indicating that OT rescued anxiety-like behavior in male mice. These results show that replenishing OT in male mice is effective against impaired anxiety-like behavior detected by the light–dark transition test (Table 1).

Another observation from our study is the presence of signs of depression in female mice in two tests. While data of tail suspension test clearly indicate genotype but no sex difference, sucrose preference test indicated specific deficit in *Chd8*^+/∆SL^ female mice only. In the current study, we measured the influence of OT on anxiety in social behavior but did not evaluate its effects on depressive phenotype in females. This is a curious question for the next step.

Rescue by OT seems in part to be due to lower plasma OT concentrations in *Chd8*^+/∆SL^ male mice. Because we have reported that circulating OT can cross the blood–brain barrier and be transported into the brain via the receptor for advanced glycation end-products [51, 52], OT is believed to act on OT receptors in brain regions, such as the hypothalamus and amygdala, to correct abnormal social behavior [53]. Therefore, it will be of interest to determine if an expression of the OT signaling system is altered in *Chd8*^+/∆SL^ mice.

In addition, it is necessary to determine whether such an OT system is present downstream of the *CHD8* gene by molecular genetic methods. It has been shown that CHD8 plays a role in the neurogenesis of the amygdala [13] and mutation of *CHD8* haploinsufficiency causes dysfunction in neuronal networks, which may result in an excitation-inhibition imbalance [53]. In *Chd8*^+/∆SL^ mice, therefore, it is possible that abnormal social behavior likely reflects impairment of the amygdala [54, 55], as a peripheral application of OT can be recruited from the blood to the amygdala in humans [56] and mice [52]. OT effects are likely due to the activation of OT-sensitive neurons in the amygdala, with respect to balancing excitability.

Plasma levels were lower in *Chd8*^+/∆SL^ mice and this difference possibly was driven by males. However, due to the limited sample amount we achieve the only tendency for genotype × sex interaction, which important limitation for our conclusion. By the way, the possible absence of change in plasma OT level in females can explain the absence of any abnormalities in maternal behavior of *Chd8*^+/∆SL^ females.

Another known issue, the results of OT measurement in plasma by immunoassay can be distorted in absence of extraction procedure due to matrix interference effects [57]. However, we used the deproteination method to remove proteins as the main source of interference, hence this possibility is unlikely [58].

Because the majority of OT neurons release OT into the blood, we speculate that in *Chd8*^+/∆SL^ mice, the release of OT from axon terminals may be attenuated. OT neurons project concomitantly to the posterior pituitary and brain regions controlling fear responses, such as the central amygdala [59, 60]. Therefore, it can be predicted that CHD8 directly or indirectly affects the central axonal release of OT.

## Conclusion

In conclusion, the observed alteration of the OT system and compensatory effect of OT on impaired social behavior in mice provides a clinically/therapeutically-relevant target for patients with ASD associated with *CHD8* mutation.

## Methods

### Animals

The deletion method of the long and short forms of CHD8 has been described previously [4]. Experiments were performed on littermates produced by WT × heterozygote (*Chd8*^+/∆SL^ or *Chd8*^+/∆L^) crosses. In the experiment, WT and *Chd8*^+*/∆SL*^ mice from the same littermates were used. All animals for the experiment were obtained from the breeding farm within the animal facility in Kanazawa University. All pups were genotyped according to previously described methods [4]. Young virgin animals (8–12 weeks) of both sexes were used for experiments. For maternal behavior experiments, primiparous lactating dams (Lactating day 3–5) were used. A male and female of each genotype were kept in a nursing cage in our laboratory under standard conditions (24 °C; 12-h light/dark cycle, lights on at 08:00) with food and water provided ad libitum. Animals were observed on a daily routine basis and if clinical signs of significant distress were observed (weight loss, reduced food intake, abnormal posture) that led to exclusion from experiment and euthanasia. All the animal experiments were performed in accordance with the Fundamental Guidelines for the Proper Conduct of Animal Experiments and Related Activities in Academic Research Institutions under the jurisdiction of the Ministry of Education, Culture, Sports, Science, and Technology of Japan and were approved by the Committee on Animal Experimentation of Kanazawa University.

### Euthanasia

After ending of behavioral experiments all mice were euthanized by inhalation of anesthesia gas (5% isoflurane in the airflow), until deep unconscious condition determined by the absence of visible breathing within one minute. Euthanasia was confirmed by followed decapitation. The method was chosen according to the recommended protocol for small rodents with a weight lower than 500 g and an age of more than 10 days.

### Open field test and social avoidance test

In the 10-min test (target absence, adaptation phase), the experimental mouse was allowed to freely explore a square-shaped arena (600 mm × 600 mm). Video tracking software (ANY-maze; Stoelting Co., Wood Dale, IL, USA) was used to measure the amount of time the experimental mouse spent in the center zone (300 mm × 300 mm), traveled distance, immobile time, and immobile time in the center zone.

Immediately after the open field test, the social-avoidance test was performed as described previously [38]. In the first phase, the mouse was returned to its home cage and then reintroduced for 10 min to the arena containing a wire mesh cage (non-social object) in the center of the arena.

In the second phase, the mouse was reintroduced into the arena for 10 min with an unfamiliar C57BL/6 mouse of the same sex in the wire mesh cage. Video tracking software (ANY-maze) was used to measure the amount of movement. For both periods (non-social object phase and social stimulus stage) traveled distance, immobile time, time in center, and immobile time in the center were measured.

### Sociability and social novelty preference test

The test was previously described by Crawley [46]. Social behavior was determined by whether a subject mouse tended to spend time with a familiar or stranger mouse in a three-chambered box. Dividing walls had doorways allowing access into each chamber. (A) Habituation. A test mouse was first placed in the middle chamber and allowed to explore for 10 min to all parts of the arena. Each of the two sides contained an empty wire cage (non-social target). (B) Sociability. An unfamiliar mouse (stranger 1; a naïve C57BL/6 mice of the same sex) was placed in the wire cage in the left chamber. In the right chamber, a non-social object was placed, and the test mouse was allowed to explore for 10 min. (C) Social novelty preference. The mouse was reintroduced to the apparatus in which the right corner was replaced with a novel naïve C57BL/6 mouse of the same sex (stranger 2). The amount of time spent in each chamber and the number of entries into each chamber were measured using a digital video system and ANY-maze software. At the end of each test, the apparatus was sprayed and wiped clean with 1% sodium hypochlorite and 70% ethanol.

Results of the experiment were determined by calculating two types of ratios.

The sociability ratio was calculated by the following formula (t Str1 – t object)/(t Str 1 + t object) where t Str1 represents the time spent in the chamber with stranger 1, and t object represents the time spent in the chamber with the inanimate non-social object.

The social novelty ratio was calculated by the following formula: (t Str2 – t Str1) / (t Str2 + t Str1), where t Str1 represents the time spent in the chamber with stranger 1, and t Str2 represents the time spent in the chamber with stranger 2.

### Light–dark transition test

The light–dark transition was measured as previously described by Crawley [42] and Lopatina [40]. The chamber consisted of two divided rooms: one was brightly illuminated (250 lx), whereas the other was dark (2 lx). A test was performed in two ways. In a direct scenario, a mouse was placed into the illuminated (light) area and allowed to move freely between the two chambers. In the reversed variant of the test, the mouse was first placed in a dark area. Each trial was recorded for 10 min using the ANY-maze video system. Latency to enter (defined by all four paws entering), time spent, entries, and distance traveled in the light chamber were recorded. Entries and time spent in the light zone were used as primary outcome measures.

### Elevated plus maze test

The elevated plus maze test was performed for five min, as described previously [39]. The maze was made of four black plexiglass arms with two open arms (67 × 7 cm) and two walled arms (67 × 7 × 17 cm) facing each other and connected by a neutral space in the center at 55 cm above the floor under dimly illuminated light (20 lx). The time spent and the frequency of entry into each arm in 5 min was automatically tracked by the camera-assisted ANY-Maze software. Time spent in open arms was evaluated.

### Tail suspension and sucrose preference tests

The tail suspension test was performed as previously described [39]. Total immobile time was measured. For the sucrose preference test, based on our previous results [37], mice were housed individually and given a two-bottle choice between distilled water and 1% sucrose solution. Sucrose preference was calculated according to formula W0 − W1/((W0 − W1) + (S0 − S1). Where S0 and W0 are the weight of bottles with sucrose solution and water before the test and S1 and W1 1 h after the test.

### Maternal retrieval behavior

The design of the parental retrieval behavior experiments was performed as described previously [39]. Five pups were randomly selected from a litter and individually placed at a site remote from the nest in the original cage. The dams were returned to the original home cage to assess pup retrieval over a 10 min period. The latency to start to retrieve pups to the nest was measured.

### Drug treatment

Mice were administered saline or OT (100 ng/100 g of body weight), ~ 0.3 ml per mice intraperitoneally (i.p.), 30 min before the start of experiments.

### Blood collection

Mice were anesthetized by i.p. injection of pentobarbitone (35 mg/kg). Blood samples (1 ml) were collected by cardiac puncture and centrifuged at 1600 × *g* for 15 min at 4 °C. Plasma samples (200–400 µl/mouse) were collected and stored at – 80 °C until use.

### Enzyme immunoassay for OT

Plasma samples were extracted by addition of a proportional solution (1:1) containing 95% acetonitrile/5% trifluoroacetic acid (0.1%). After vortexing for 30 s and centrifugation at 15, 000 rpm, supernatants were evaporated in a Centrivap Vacuum Concentrator (Labconco, Kansas City, MO, USA). The resulting pellets were dissolved in assay buffer. Determination of OT was performed using a 96‐plate commercial OT ELISA kit (Enzo Life Sciences, Farmingdale, NY, USA), as described previously [18, 43].

### Statistical analysis

GraphPad Prism 6 (GraphPad Software, San Diego, CA, USA) was used for statistical analysis. Data are expressed as mean ± S.E.M. Comparisons were evaluated between male and female mice of two genotypes (WT or *Chd8*^+/∆SL^). In some experiments, mice treated by OT or saline were compared. Wild-type mouse used as a negative control and Chd8^+/∆SL^ mouse as an experimental group. In experiments with the administration of OT, saline-treated groups were considered as negative control groups. Results of genotyping were used as criteria of inclusion to the control or experimental group. No specific criteria for exclusion were used. A single animal was considered as experimental unit. The total number of used animals in behavioral tasks without pharmacological intervention: WT males: 14, WT females: 13, *Chd8*^+/∆SL^ males: 10, females: 8. While for each experiment the mouse was introduced only once, some of the tested animals used in different behavioral experiments. In pharmacological experiments, maternal behavior, and OT measurement study all individual mice we used just once. Total amount of used mouse for OT measurement: 33, light–dark box experiment: 60; 3-chamber test: 83; for maternal test: 10. The experiments were performed and analyzed by researchers who were blind to treatment and genotype. Two-way analysis of variance (ANOVA) (genotype × sex) was used to analyze the statistical differences among the groups by GraphPad Prism 8.4. If sex–genotype interaction was observed, multiply comparison followed by Bonferroni correction was applied. Data were considered statistically significant when the probability of a type I error was < 0.05. In pharmacological experiments, Three-way ANOVA (genotype × sex × treatment) was performed. Multiple comparisons followed by Tukey correction were performed. In Open Field and Social Avoidance Task experiment four outcome measures were used, hence to avoid type II error, Bonferroni correction for multiply comparisons was applied in all cases.

## Supplementary Information

**Additional file 1: Fig. S1.** Behavior of wild-type (WT, blue) and Chd8^+/∆SL^ (Chd8^+/-^, red) mice in the open field test and social avoidance test. Travelled distance in open field (**a**), immobility time in center during open field task (**b**), Travelled distance in non-social phase of social-avoidance task (**c**) time in center zone (**d**) and immobility time in the center zone. Traveled distance in social object phase of social avoidance task (**f**). Columns with superscript letters (a) are represent absent of difference.

**Additional file 2:** **Figure S2.** Maternal retrieval Behaviour. N = 5. Time to complete retrieving 5 pups for to the nest. In all cases dams retrieved all pups directly to the nest place of cage.

**Additional file 3:** **Figure S3**. Effects of oxytocin on 3-chamber Performance. Sociability phase for males (a) and females (b) and Social Preference phase for males (c) and females (d).

## Data Availability

The datasets used and/or analysed during the current study are available from the corresponding author on reasonable request.
