# Supplementary figures and images for: Oxytocin ameliorates impaired social behavior in a Chd8 haploinsufficiency mouse model of autism
Source: BMC Neurosci. 2021 May 1;22:32. doi: 10.1186/s12868-021-00631-6 (PMC8088024; doi:10.1186/s12868-021-00631-6)

## Slide 1
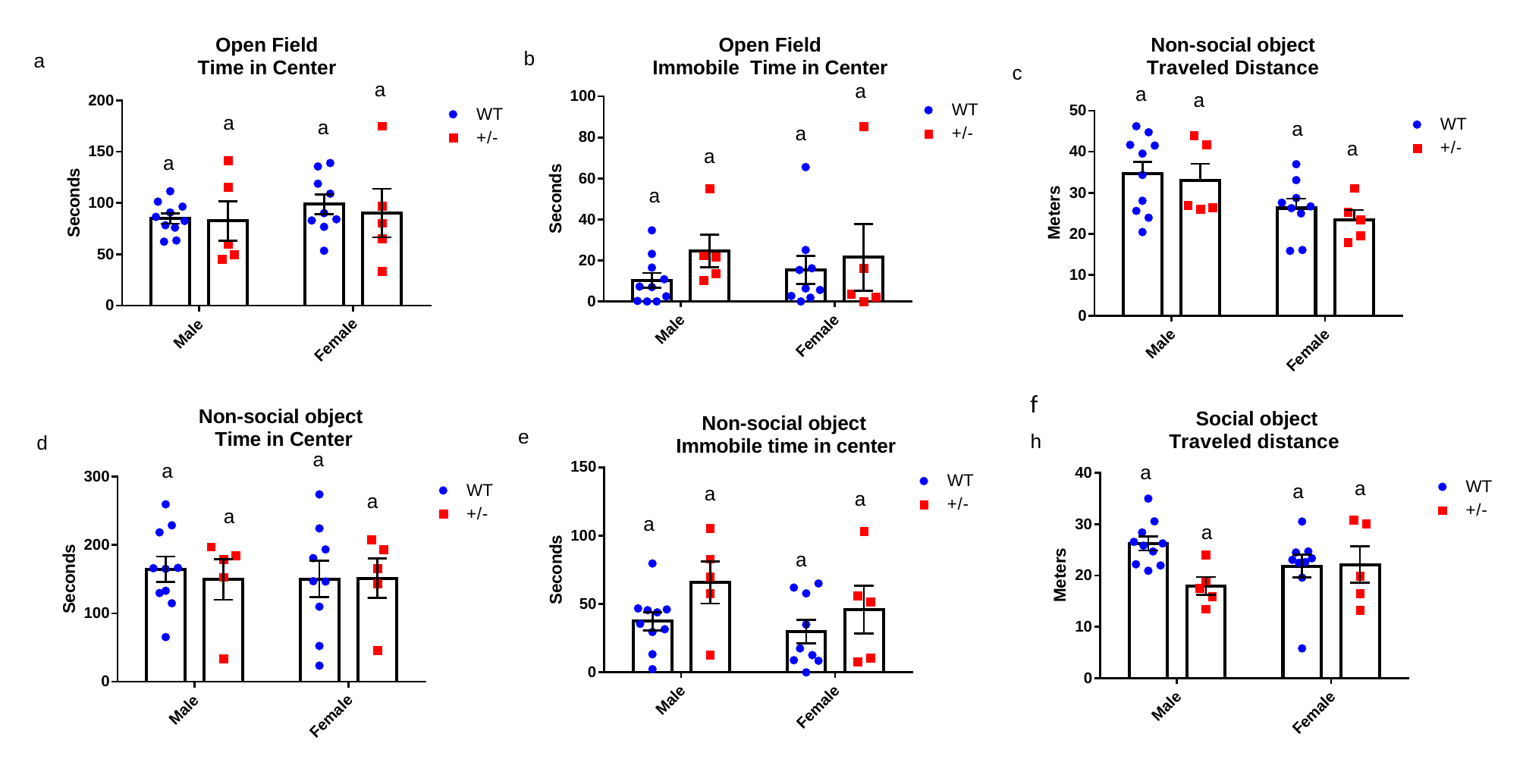

f

Supplement: Supplementary file 1 — Additional file 1: Fig. S1. Behavior of wild-type (WT, blue) and Chd8+/∆SL (Chd8+/-, red) mice in the open field test and social avoidance test. Travelled distance in open field (a), immobility time in center during open field task (b), Travelled distance in non-social phase of social-avoidance task (c) time in center zone (d) and immobility time in the center zone. Traveled distance in social object phase of social avoidance task (f). Columns with superscript letters (a) are represent absent of difference. [file 12868_2021_631_MOESM1_ESM.pptx]

## Slide 1
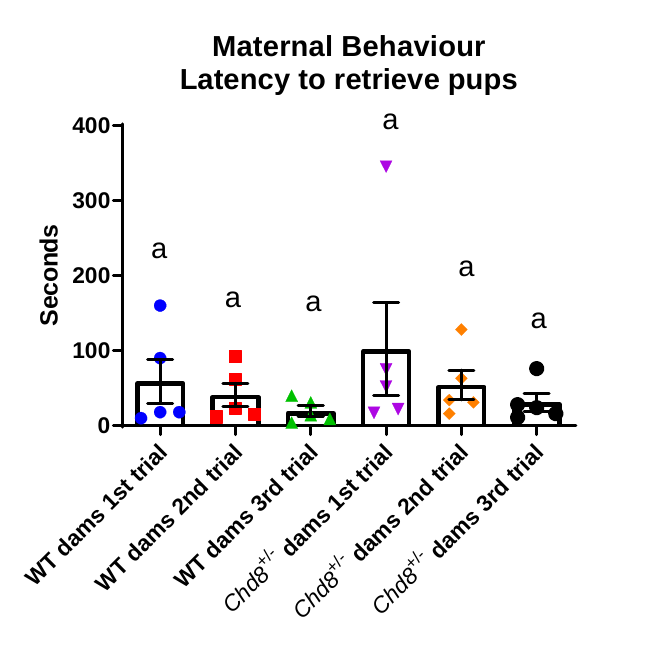

Supplement: Supplementary file 2 — Additional file 2: Figure S2. Maternal retrieval Behaviour. N = 5. Time to complete retrieving 5 pups for to the nest. In all cases dams retrieved all pups directly to the nest place of cage. [file 12868_2021_631_MOESM2_ESM.pptx]

## Slide 1
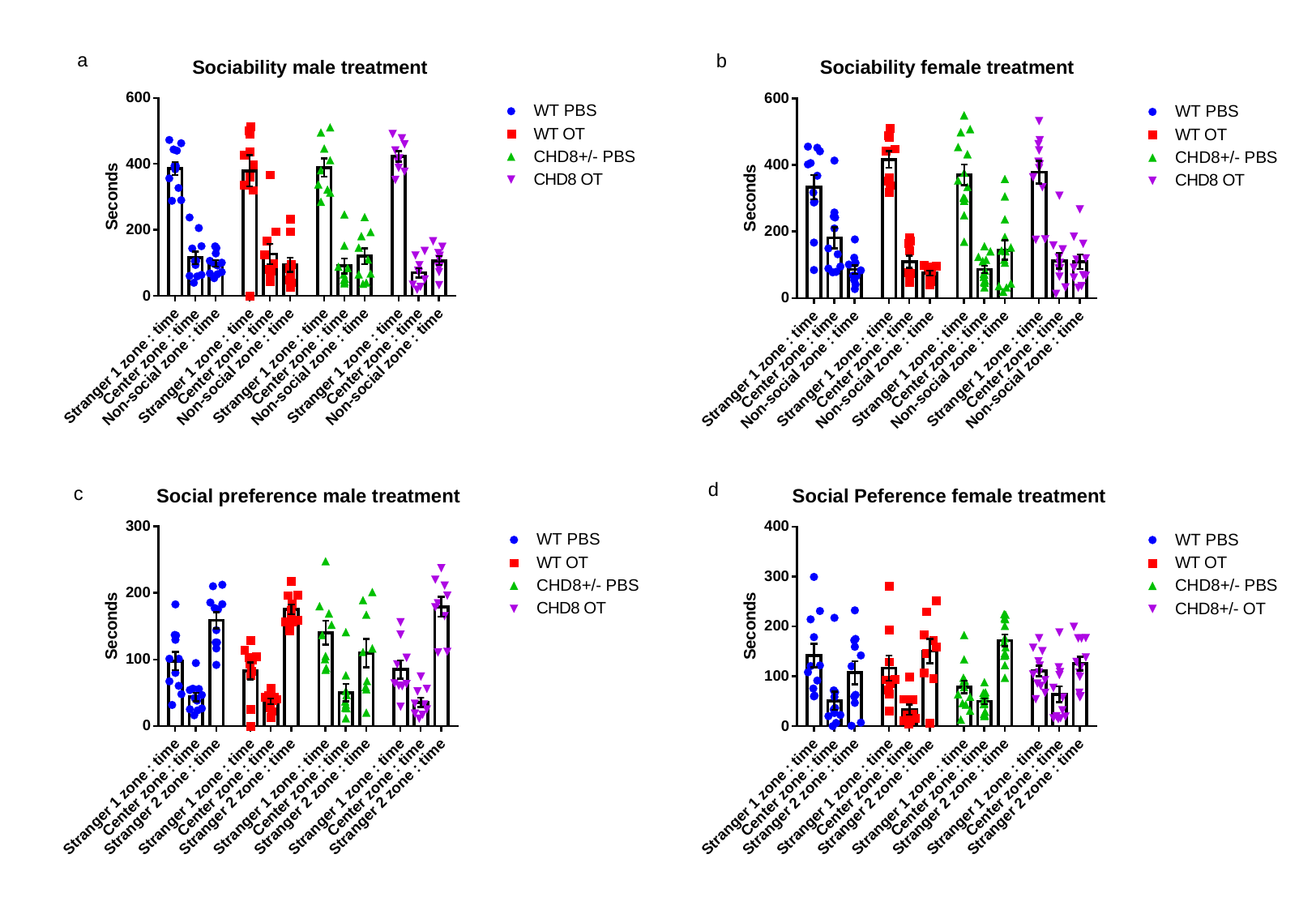

Supplement: Supplementary file 3 — Additional file 3: Figure S3. Effects of oxytocin on 3-chamber Performance. Sociability phase for males (a) and females (b) and Social Preference phase for males (c) and females (d). [file 12868_2021_631_MOESM3_ESM.pptx]
